# The evolution of dam-litter microbial flora from birth to 60 days of age

**DOI:** 10.1186/s12917-022-03199-3

**Published:** 2022-03-11

**Authors:** Angela Del Carro, Michela Corrò, Alessia Bertero, Barbara Colitti, Penelope Banchi, Luigi Bertolotti, Ada Rota

**Affiliations:** 1 Department of Veterinary Sciences, University of Turin, Turin, Italy; 2 Istituto Zooprofilattico Sperimentale delle Venezie, Legnaro (Padua), Italy

**Keywords:** Puppy microbiota, Bacterial colonization, Meconium, Microbial flora, Litter bacterial flora

## Abstract

**Background:**

Early bacterial colonization in puppies is still a poorly understood phenomenon. Although the topic is of considerable interest, a big gap in knowledge still exists on the understanding of timing and features of neonatal gut colonization. Thence, the purpose of this study was to evaluate the relationship between dam and litter microbial flora, in vaginally delivered puppies, from birth to two months of age. Bacteria were identified using MALDI-TOF, an accurate and sensitive method, and cluster analysis of data provided a new insight on the investigated topic.

**Methods:**

Six dam-litter units of two medium size breeds were enrolled in the study. Vaginal and colostrum/milk samples were collected from dams after delivery and 48h post-partum, while rectal samples were taken from dams and puppies after delivery and at day 2, 30 and 60 (T2, T30 and T60, respectively) post-partum. Bacterial isolation and identification were performed following standard techniques, then the data were analyzed using a new approach based on bacterial genus population composition obtained using a wide MALDI-TOF screening and cluster analysis.

**Results:**

Forty-eight bacteriological samples were collected from the dams and 145 from their 42 puppies. Colostrum/milk samples (*n* = 12) showed a bacterial growth mainly limited to few colonies. Staphylococci, Enterococci, *E. coli*, *Proteus* spp. were most frequently isolated. All vaginal swabs (*n* = 12) resulted in bacteria isolation (medium to high growth). Streptococci, Enterococci, *E. coli* were the most frequently detected. *E. coli*, *Proteus mirabilis*, *Enterococcus* spp., *Streptococcus* spp. were often obtained from dams’ and puppies’ rectal swabs. Clostridia*,* not isolated in any other sampling site, were rarely found (*n* = 3) in meconium while they were more frequently isolated at later times (T2: *n* = 30; T30: *n* = 17; T60: *n* = 27).

Analysis of the bacterial genus pattern over time showed a statistically significant reduction (*P* < 0.01) in the heterogeneity of microbial composition in all time points if compared to birth for each dam-litter unit. These results were confirmed with cluster analysis and two-dimensional scaling.

**Conclusion:**

This novel data analysis suggests a fundamental role of the individual dam in seeding and shaping the microbiome of the litter. Thus, modulating the dam’s microbiota may positively impact the puppy microbiota and benefit their health.

**Supplementary Information:**

The online version contains supplementary material available at 10.1186/s12917-022-03199-3.

## Background

Few studies have been conducted to increase the understanding of the early bacterial colonization in puppies and to explain the role of the dam's microbial flora.

In humans, neonatal gut colonization depends on multiple factors, including delivery mode and type of feeding (breastfeeding *vs* formula feeding). In case of vaginal delivery, the newborn gastrointestinal tract is colonized by maternal vaginal and gut bacteria [1] while cesarean-born babies have a gastrointestinal microbiota similar to maternal skin and oral microbiota [2]. Breast feeding or formula milk have a significant influence on early microbial colonization. Breast milk is a source of nutrients and bioactive compounds that promote the growth and development of the immune system in babies, and its unique microbiome has recently been described [3–5]. Breast milk is the other most significant source of microbes for newborn babies and infants after vaginal birth [6]. The presence of an entero-mammary pathway has been demonstrated, and translocation from the intestine to the mammary ducts was observed for some bacteria [7]. Other factors such as antibiotic exposure, age, mother’s diet and genetic background [8] affect neonatal gut colonization and bacterial population composition [9]. At weaning, the dietary change, from breast milk to solid food, causes a modification in the relative abundance of the bacterial genera in the gut microbiota of infants [10, 11].

In dogs, timing of initial bacterial colonization, whether during fetal life or at birth, and newborn puppy microbiota have been the object of recent investigations [12, 13]. The presence of a microbial flora at birth [13] and the effect of delivery mode on meconium microbiota [12] have been described using traditional cultural methods. Newborn puppies have a very limited capacity of movement, and they fully depend on maternal interaction. Maternal care and nursing expose them to the dam’s microbial flora [14]. Canine neonates are also in contact with environmental bacteria, although they live in a restricted environment represented by the nest and whelping box. Daily interactions with the breeder represent a further source of microorganisms.

As previously seen in humans [15], the type of diet has a significant impact on shaping the microbial gut composition of canine neonates and canine milk plays a key role in colonization and modulation of gut microbial population of puppies. It is known that the dam’s milk serves to transfer nutrients and immunoglobulins to the offspring [16] and *Lactobacillus* species [17].

The canine neonatal and early pediatric fecal microbiome has also been characterized through next generation sequencing (NGS) techniques [18] and has been compared with the dam’s fecal [18] and milk [19] microbiota.

The aim of this study was to assess the relationship between dam and litter microbial flora, in vaginally delivered litters, from birth until two months of age. Considering a wide characterization of bacterial population with MALTI-TOF analysis, we evaluated the similarity among dams and puppies by cluster analysis. This new data analysis approach can help us to better understand the role of the microbial flora of the dams in shaping their offspring gut microbiota.

## Results

Forty-two puppies were born. Stillbirths were six, all due to prolonged parturition, while a Lagotto Romagnolo (LR) puppy died 7 days (lower-than-average birth weight) and another one 14 days after birth. Necropsy of stillborn and dead puppies always excluded Canine Herpesvirus and *Brucella* spp. infections. Appenzeller Cattle Dog (ACD) puppies’ mean birth weight was 410±0.05 g while LR puppies’ mean birth weight was 240±0.05 g. The growth curves of the puppies of the two breeds agreed with the breed and with the breeding facility experience.

Forty-eight bacteriological samples were collected from the dams and 145 from their puppies, at the different sampling times.

No colostrum or milk samples (*n* = 12) resulted sterile, even if bacterial growth was frequently limited to few colonies. Staphylococci were isolated from colostrum and milk (*Staphylococcus pseudintermedius* from three samples and coagulase negative staphylococci from other two). Except for three samples, Enterococci were always isolated and in one case only *Aerococcus* spp. (*A. viridans*) was detected. Among Gram-negative bacteria, *Escherichia coli* was isolated from the 50% of the colostrum samples and from 17% of milk samples. *Proteus* spp. (*P. mirabilis*) was isolated from 50% of colostrum samples and from 33.3% of milk samples. From both matrices there was a single isolation of *Enterobacter* spp*.* and of *Klebsiella* spp*. (K. pneumoniae). Pseudomonas* spp. and *Pshychrobacter* spp. were isolated from a single colostrum sample.

Vaginal swabs (*n* = 12) always resulted in bacteria isolation, generally showing medium to high growth. Three dams harboured Streptococci (*Streptococcus canis*); one of them was also the one from which *S. pseudintermedius* was isolated; from another dam *S. saprophyticus* was isolated. More samples resulted in growth of Enterococci (*E. faecalis*, *E. fecium*). Among Gram negative bacteria, *E. coli* was more frequently isolated (seven samples), followed by *Klebsiella* spp. (*K. pneumoniae*) and *Enterobacter* spp. (*E. cloacae*) (two samples); *Proteus* spp. (*P. mirabilis*) and *Leclercia* spp. (*L. adecarboxylata*), one sample each.

**Fig. 1:**
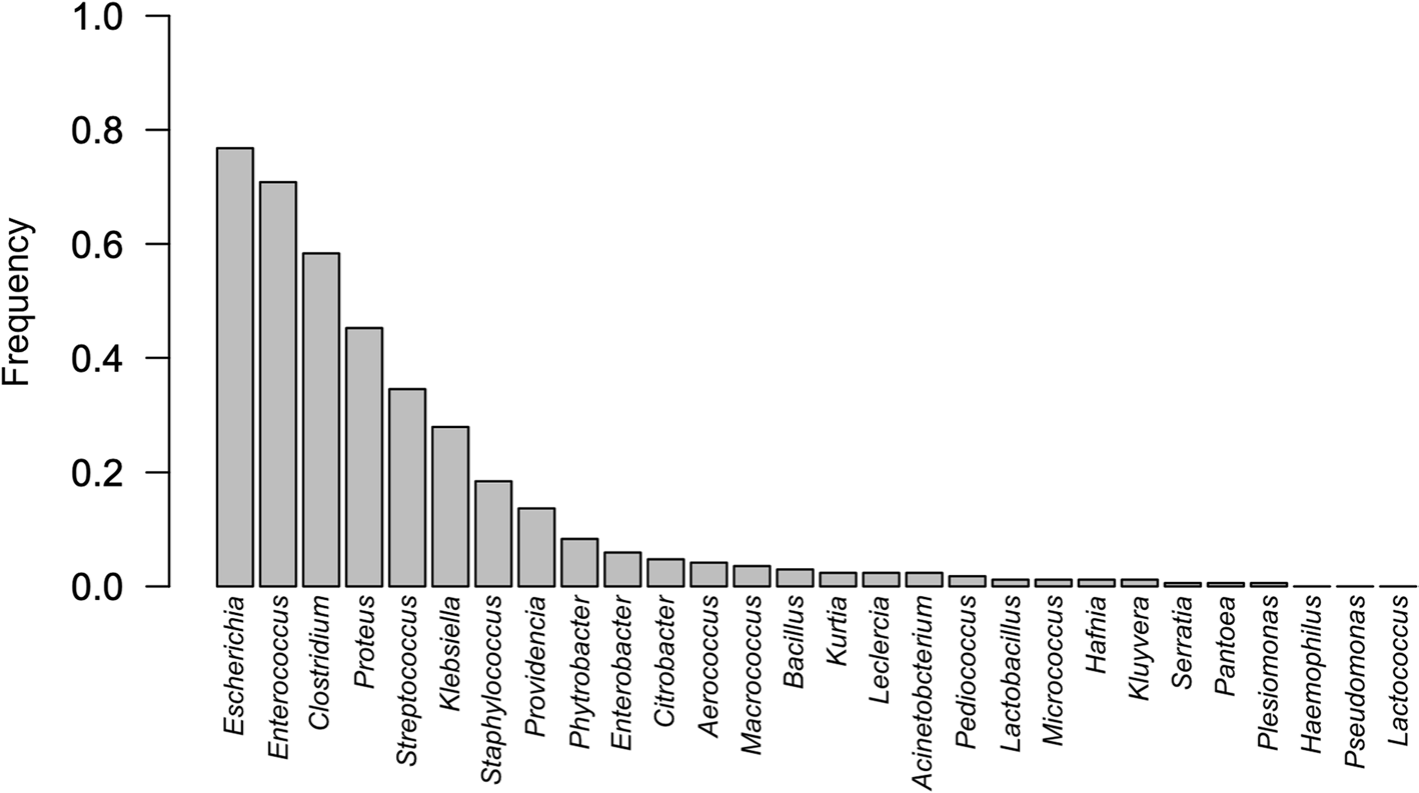
Isolation frequency of the different bacteria from the rectal swabs of the six dams (*n* = 24) and their 42 puppies (*n* = 145)

The bacteria isolated from dams’ and puppies’ rectal swabs are listed in Fig. 1, where isolation frequencies are also reported. *Escherichia coli*, *Proteus mirabilis*, *Enterococcus* spp., *Streptococcus* spp. were more frequently present, together with *Clostridium perfringens*, which was not isolated in any other sampling sites.

**Fig. 2:**
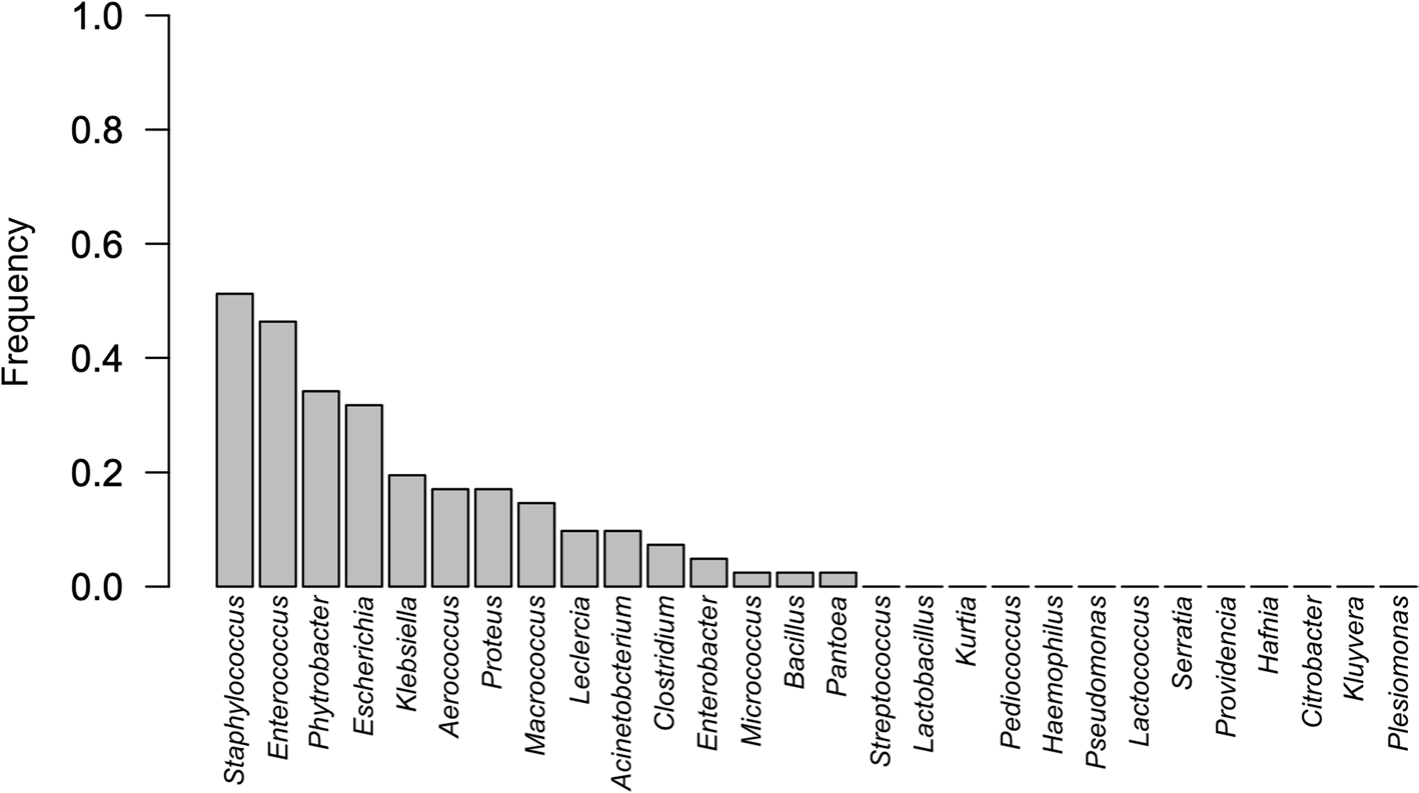
Isolation frequency of the different bacteria from meconium (42 puppies: rectal swabs at birth)

Figure 2 shows the frequency of isolation of bacteria grown from meconium samples collected at birth (T0). Clostridia (*Clostridium perfringes*) represented a rare finding in meconium and was indeed isolated only from three puppies of the same litter. In contrast, as for the rectal swabs performed on puppies on day 2, 30 and 60 post-partum, Clostridia growth was observed in 30, 17 and 27 specimens, respectively. The Gram-negative bacteria *E. coli* and *Proteus* spp. showed a lower frequency of isolation in meconium while *Psychrobacter* spp. was found in more than 30% of the samples.

## Data analyses

**Fig. 3:**
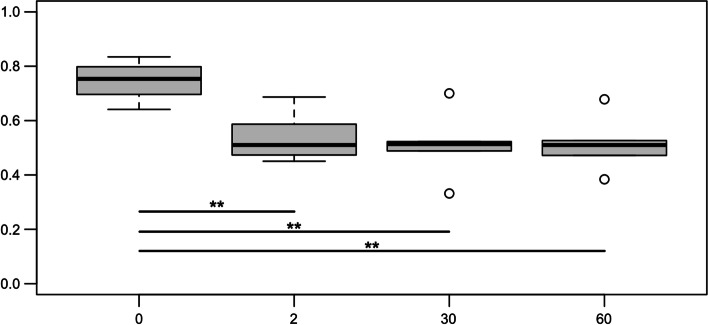
Distribution of within-family heterogeneity trough time. Significant difference is reported (** Wilcoxon *p* < 0.01)

The codified 1/0 pattern was assigned to each sample, according to the presence or the absence of a given bacterial genus (Supplementary Fig. 1). Based on the pattern detected by MALDI-TOF, the within-family heterogeneity decreased through time, showing a statistically significant reduction in all time points if compared to birth. The reduction was confirmed also considering rectal swabs only, to avoid the possible bias given by different samples matrices (Wilcoxon test results are reported in Fig. 3).

**Fig. 4:**
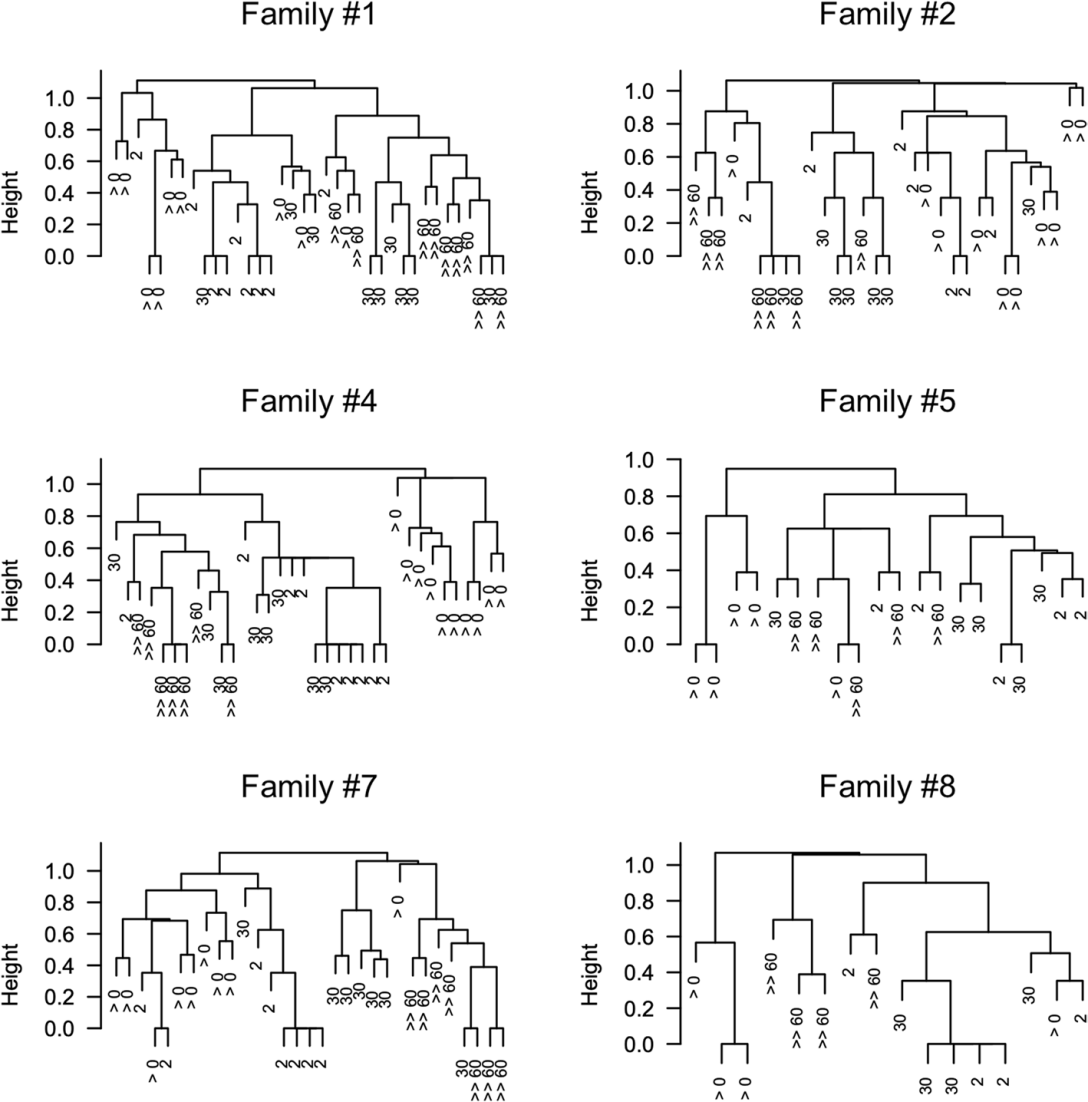
Single family complete linkage dendrograms. Samples collected at T0 (>) and T60 (>>) are highlighted

Complete-linkage cluster analysis based on the similarity (Pearson’s correlation index) among bacterial population composition patterns of rectal swabs showed how samples collected at T0 and T60 tend to cluster in two separate groups. As depicted in Fig. 4, T60 samples are closer and more similar compared to T0 samples (smaller height value of common nodes), confirming previous analyses.

**Fig. 5:**
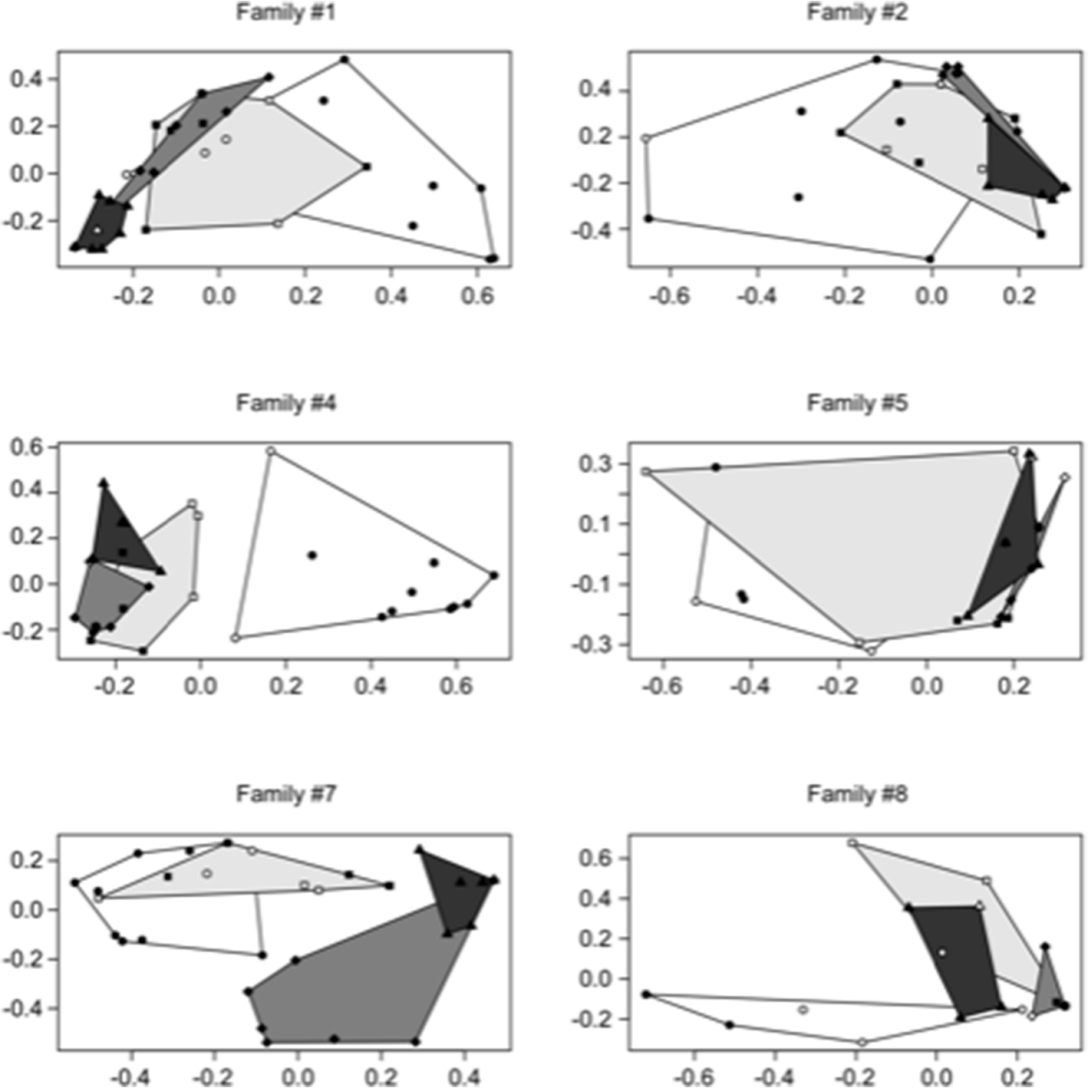
Single family two-dimensional scaling. Samples collected at the same collection time are grouped by a polygon (T0: circle/white polygon; T2: square/light gray polygon; T30: diamond/gray polygon; T60: triangle/dark polygon). Dams are reported by white symbols, puppies by black symbols

**Fig. 6:**
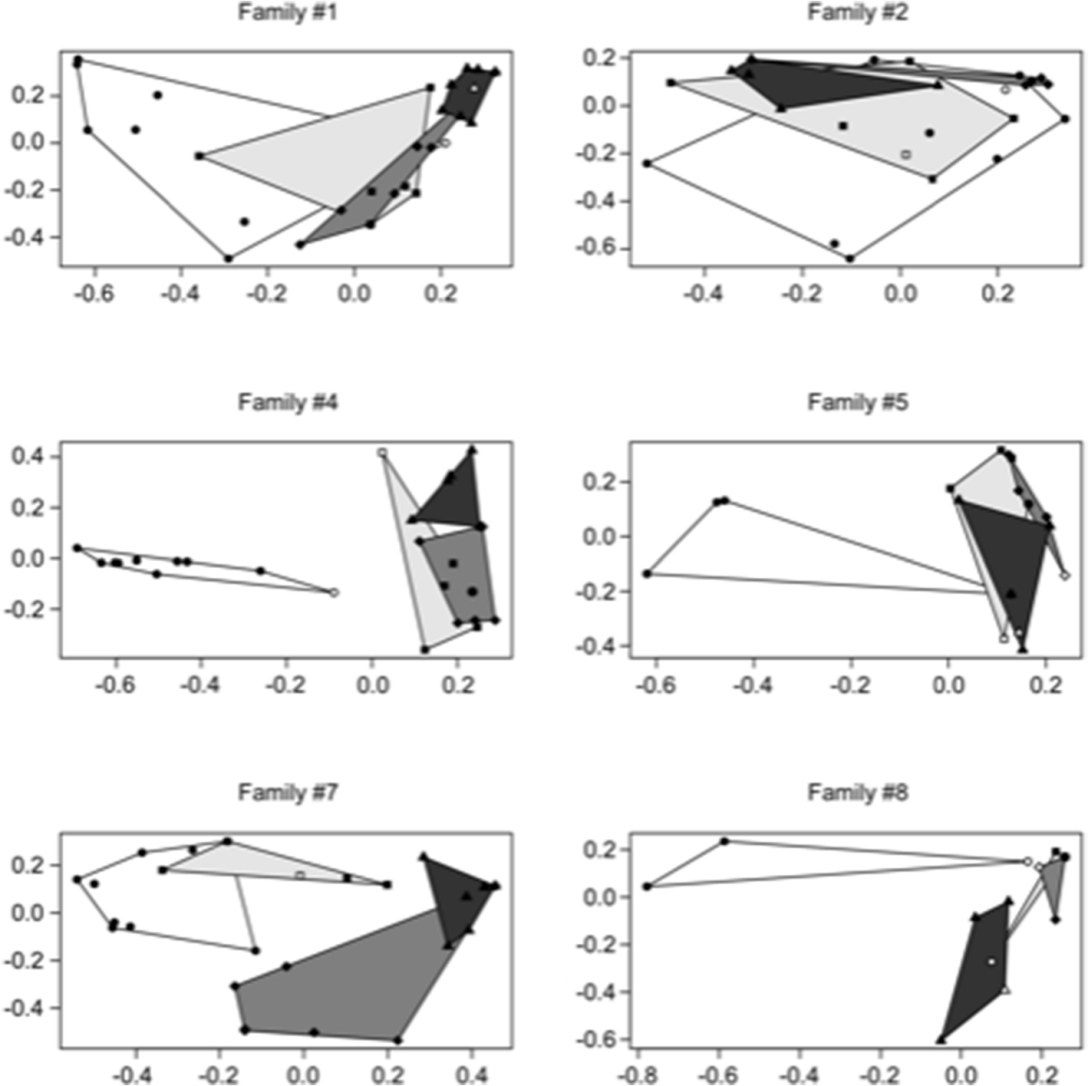
Single family two-dimensional scaling. Rectal samples collected at the same collection time are grouped by a polygon (T0: circle/white polygon; T2: square/light gray polygon; T30: diamond/gray polygon; T60: triangle/dark polygon). Dams are reported by white symbols, puppies by black symbols

This aspect is also confirmed if a two-dimensional scaling reconstruction is used, including both all the samples (Fig. 5) and rectal swabs only (Fig. 6): 2D approach confirmed the decrease in microbiome diversity and change in clustering pattern. More in detail, both the area and the position of samples within the 2DS space changed at the different experiment time points. It is clear how microbial genus composition tends to be less heterogeneous at the end of the experiment, even if all the families started from a highly divergent scenario. Areas covered by samples collected at the first time point are significantly wider than the areas covered by samples at T30 and T60 (Wilcoxon test < 0.01 in both the comparison).

## Discussion

Traditionally, the establishment of neonatal gut microbiota is believed to start at birth, as soon as the amniotic membrane breaks; the newborn infant is exposed to bacteria from the mother’s vaginal and fecal microflora during birth and the exposure to external environmental bacteria takes place immediately after [20]. Furthermore, maternal breast milk promotes the colonization and maturation of the infant gut flora of neonates [21]. Nevertheless, the early colonization process shows specific features in dogs, because the amniotic membrane is generally intact when puppies are born and the bitch tears it with her teeth. As a consequence, the birth canal does not seem to be the major source of the neonatal flora in this species [14, 22] and puppies are therefore exposed both to the microbial flora of the bitch and of the local environment.

Recent investigations have challenged the dogma of a sterile fetal environment and suggested the possibility of an in-uterus seeding of the microbiota, in humans, and dogs, among other animal species [13, 23, 24].

It is then not surprising to isolate bacteria from puppies’ meconium [12, 13]. However, the extent to which maternal flora and environmental bacteria contribute to the puppies’ gut microbial flora has not been fully investigated in dogs. Our results provide a novel picture on the bacterial rectal flora of the dam, shaping that of the litter over time. We detected a higher similarity, expressed as Pearson’s correlation index (see Methods for details), in microbial flora within the single dam-litter unit than among the different units, even if the dams belonged to the same kennel. The second observation is the reduction over time of the diversity of the isolated bacteria, with the composition of the distal gut microbial flora showing a trend towards a defined composition. All the dams and puppies included in our study shared the same environment and the same diet, thus the effect we see in the dam-litter units is mainly due to the individual microbiota profile. Accordingly, when Vilson et al. [19] studied German Shepherd dogs using NGS techniques, they demonstrated that the fecal microbiota of 7 weeks old puppies was more similar to that of their mothers than of unrelated bitches and that littermates had a more similar fecal microbiota compared to unrelated dogs. The dam-litter similarity could also be explained by the dam’s behavior of eating her puppies’ stool, thus reciprocally conditioning their microbiota profile [19].

Similar observations can be drawn from another study that evaluated the progression of puppy fecal microbiota beginning at two days of age, by means of sequencing technologies [18]. From day 2 to 21 after birth an increased microbial diversity and richness was detected; the highly variable bacterial profile was more stable by day 42, although still different from that of the dam. At 56 days of age the fecal microbiota in puppies resulted significantly different from that of their mothers; although they clustered separately, the microbial communities had a lower distance and showed more similar species [18].

By 24 hours after birth, numbers of bacteria in the distal portion of the newborns’ colon were comparable to those of the dams [25]. After the initial colonization, further changes involved only shifts in the relative proportions of the various populations that constitute the bacterial ecosystem of the gastrointestinal tract. Beginning from 1 day-old newborns to 63 day-old puppies 1 week after weaning, an increase was observed in the relative proportion of anaerobic bacteria in the distal portion of the colon, in which anaerobic conditions predominate [25].

From weaning to adult age changes occur in the gut microbiota, as observed in German Shepherd dogs that showed a fecal microbiota of significantly different composition between 7 weeks and 15-18 months of age [19]. A higher relative stability of the gastrointestinal microbiota is expected when reaching adult life.

A breed effect was observed on the fecal microbiota composition in dogs [26]; however, although the dams are evenly distributed between the two breeds in our study, the number of animals is too low to assess a breed difference. Besides, differently from the study by Reddy et al. [26], we adopted culturing methods: compared to culture-independent NGS investigations, traditional techniques have the advantage to discriminate between living and dead microorganisms and to identify the genus when not the species. In this regard, MALDI-TOF MS-based bacterial identification allows the characterization of unique mass spectra (protein fingerprints) which are typical of each microorganism, providing robust and reliable results. The limit of bacteria culture is given by the risk to miss several microorganisms, particularly those that do not find their growth needs satisfied in culture, so that genomic observations find an extraordinarily larger number of taxonomic units [27].

When comparing rectal swabs at birth with later puppies’ rectal samples and with the dams’ rectal samples in our study, the most evident results are the almost complete absence of *Clostridium* spp. and the low frequency of isolation of enteric bacteria (*E. coli*, *Proteus* spp., *Enterobacter* spp.) in meconium. Previous observations on meconium bacterial flora were made on samples collected immediately after colostrum intake to stimulate defecation, thus allowing time for possible colonization [28] but, notwithstanding this different approach, in this study Clostridia were not isolated accordingly [12]. Among enteric bacteria, *Proteus* spp. was not found while *E. coli* was isolated, but the relative frequency of isolation is not reported [12]. The findings in the distal portion of the colon of 1-day-old puppies euthanatized after suckling [25] are not fully comparable with our findings at birth. Numbers of Clostridia resulted low at day 1, increased at day 21 and decreased thereafter, reaching adult prevalence; enteric bacteria followed a declining curve as well, whereas the numbers of Lactobacilli reached the maximum at day 63 [25].

In all the investigations on meconium microflora, the possibility of contamination cannot be thoroughly ruled out because birth is not a sterile process and bacteria colonization has been demonstrated to begin immediately and occur rapidly. Gastrointestinal colonization is a fast process and by 24 hours after birth, the number of bacteria in the various tracts were found to be comparable to those of the dams [25].

Our observations suggest that there is a remodeling of the initial bacterial populations towards a decreasing heterogeneity, as observed within the dam-litter units over time. The restriction of variability that we observed occurred along weaning, when the gut microbiota may begin to increase the similarity to a young-adult condition.

Breast milk, after the vaginal and the gastroenteric tracts, is the second most significant source of microbes for newborn babies and infants after vaginal birth [6]. In dogs, colostrum is secreted by the mammary gland during the first 2 days post-partum and has a crucial importance for the immunity of newborns, containing the immunoglobulins that do not cross the canine placenta [16]; it supplies energy and contributes to the digestive tract maturation, also being a source of microorganisms for the gut microbiome.

Isolation of bacteria from colostrum has been reported in dogs [29], whereas the presence of bacteria in canine milk from healthy bitches has long been known [30]. We chose to collect the mammary secretion without previous disinfection because we wanted to have the picture of the bacteria that enter the puppies’ mouth when suckling. The samples that we milked could then be colonized by skin bacteria besides by bacteria living in the nipple ducts. The limited number of samples allows for a descriptive analysis only. Data suggest a reduction of enteric bacteria (*E. coli*; *Proteus* spp., *Enterobacter* spp.) in mammary secretion from birth to day 2, likely reflecting the highly contaminated environment of the day of parturition. The colostrum/milk contribution to the canine newborn gut microbiota is not known. What we do understand from a previous study is that oral and anal aerobic flora of 24-36 hours old puppies vaginally delivered are correlated to milk, vaginal and oral flora of the mother [31]. In our study, the decrease in variability among bacteria and the higher similarity within dam-litter units rather than among them was confirmed also when including colostrum, milk and vaginal samples.

## Conclusions

Our work increases the knowledge of the normal distal gut microbial flora at birth in dogs and of the normal bacterial flora encountered by puppies in their first suckling, immediately after birth. The novel data analysis approach has led to a very interesting picture that confirms the utmost importance of the maternal microbiota in shaping the gut microbiota of her litter. Each dam has an individual microbiota profile that influences her litter gut microbiota assembly, which leads to the conclusion that the dam seeds the initial bacterial populations. This means that it could be useful to act on the dams’ microbiota to positively condition the microbiota of their offspring particularly of their gut microbiota, which plays a key role in the function of the immune system and on future health.

## Methods

### Animals

Six dam-litter units of two medium size breeds, ACD and LR, were included in the study. The dams were three ACD, aged 5.6±1.2 years (mean ± standard deviation -SD-) and weighing 24.9±1.9 kg, and three LR, aged 4.9±3.1 years and weighing 13.1±1.1 Kg. A single LR dam was primiparous while all the others had whelped 2-5 times (Table 1).

**Table 1:** ID, breed, age, weight and parity of the bitches included in the study

**Dam ID**	**Breed**	**Age (years)**	**Weight (Kg)**	**Parity**(***N***)	**Puppies**(***N***)	
					**Alive**	**Stillborn**
A01	ACD	6.5	25.6	3	8	0
A02	ACD	4.2	22.8	2	6	3
A05	ACD	6	26.4	3	4	0
		**5.6 ± 1.2**	**24.9 ± 1.9**			
A04	LR	5.6	12.5	4	8	1
A07	LR	1.5	13.6	0	7	1
A08	LR	7.6	12.4	5	3	1
		**4.9 ± 3.1**	**13.1±1.1**	**2.8±1.7**	**36**	**6**

*ACD* Appenzeller Cattle Dog, *LR* Lagotto Romagnolo. Mean values ± SD in bold. The number of the puppies born alive and dead is reported in the last two columns

The bitches were housed in the same breeding kennel and fed with the same commercial food (MONGE Natural Superpremium Medium Adult Rich in Chicken®, Monasterolo di Savigliano, Cuneo, Italy). Over the two-week period before the expected parturition date, the dams were gradually shifted towards a dry balanced diet for growing medium size dogs (MONGE Medium Puppy & Junior Rich in Chicken®, Monasterolo di Savigliano, Cuneo, Italy). Food quantity was calculated based on FEDIAF guidelines-2019 [32].

All pregnancies were uneventful, and the bitches received only the usual deworming treatment (1 mg/Kg milbemycin oxime and 10 mg/Kg praziquantel). No antimicrobials or other drugs or supplements were administered. All the bitches delivered naturally. The number of delivered puppies is shown in Table 1.

Puppies suckled their dam’s milk until four weeks of age, when weaning began. The weaning diet consisted of the same solid food for feeding the dam and was administered until two months of age. The puppies were weighed at day 0, 2, 30 and 60. In case any puppy died, necropsy was performed.

The study was approved by the Ethical committee of the Department of Veterinary Sciences of the University of Turin (Approval number 2200, 24/09/2019). Written informed consent was obtained from the dog breeder.

### Sample collection

*Dams* - Vaginal samples were collected using a sterile nylon flocked swab (ESwab, 480CE, Copan Italia Spa, Brescia, Italy) inserted carefully into the vagina, using a sterile guide, in a craniodorsal direction and gently rotated for 30 s. Vaginal samples were collected after delivery of the last puppy and 48 hours post-partum.

Bulk colostrum and milk samples were taken milking each teat into a sterile conical test tube (Cellstar Tubes-Greiner Bio-One, IT), without previous disinfection of the area. A sterile nylon flocked swabs (ESwab, 480CE, Copan Italia Spa, Brescia, Italy) was plunged into the sample. Collection took place immediately after delivery of the last puppy (colostrum) and 48 hours post-partum (milk).

Rectal samples were collected using a sterile nylon flocked swab (ESwab, 480CE, Copan Italia Spa, Brescia, Italy) introduced in the rectum, after delivery of the last puppy and at day 2, 30 and 60 post-partum.

*Puppies* - Rectal samples from puppies were collected with a sterile mini nylon flocked swab (ESwab, 484CE, Copan Italia Spa, Brescia, Italy) immediately after puppy resuscitation and before any interaction with the dam (i.e., licking, first colostrum intake). The other rectal samples were collected at day 2, 30 and 60. In case of stillborn puppies, when possible, rectal samples were collected at birth.

All swabs were placed into modified liquid Amies medium (ESwab Copan Italia Spa, Brescia, Italy). All samples were aseptically collected by a single operator, and they were immediately sent to the laboratory of the Istituto Zooprofilattico Sperimentale delle Venezie (Legnaro, Italy), in refrigerated boxes, and processed within 48 hours.

### Bacteriological examination

Bacterial isolation was performed according to standard laboratory culture techniques. Briefly, each swab was diluted in 1 ml of nutrient broth (HIB, Heart Infusion Broth, Conda, Madrid, Spain). Ten and 100 ml of bacterial suspension were respectively inoculated into solid media and broths, as described below.

Search for aerobic microorganisms was conducted using nutrient medium (BA, Blood Agar Base n° 2, Biolife, Milan, Italy, with 5% defibrinated sheep blood, Allevamento Blood, Teramo, Italy), nutrient broth (HIB), and selective Enterobacteriaceae medium (McConkey agar, Oxoid, Basingstoke, UK), Bile-Esculin Azide Agar (Conda, Madrid, Spain). Cultures were inoculated and incubated at 37°C±1°C in aerobic conditions.

Search for anaerobic microorganisms was conducted using nutrient medium (BA), selective medium for *Clostridium perfringens* (TSC Agar Base, Biolife, Milan, Italy) and Fluid Thioglycolate medium (Liofilchem, Roseto degli Abruzzi, TE, Italy). Cultures were inoculated and incubated at 37°C±1°C under anaerobic conditions.

Culture media were checked at 24 or 48 hours depending on the aerobic or anaerobic conditions; in case of absence of bacterial growth on the plates and of turbidity in the nutrient broths, the plates were respectively re-incubated for further 24 or 48 hours, under the same conditions and broth seeding was performed as previously described.

All the microbial colonies grown on the first isolation plates were counted. Based on the number of colony forming units (CFUs), growth was classified as Low (1–10 CFU/10 μL), Moderate (11–30 CFU), or High (≥31 CFU).

Bacterial genus identification was phenotypically performed by macroscopic observation of colonies, Gram stain reaction, cellular morphology observation, growth on selective medium, catalase, oxidase, and mobility test; on catalase positive Gram-positive cocci, coagulase tube test was also performed.

Species identification was performed by MALDI-TOF MS: Microflex LT instrument (MALDI Biotyper, Bruker Daltonics) equipped with FlexControl software (version 3.3, Bruker Daltonics).

### Data analysis

To describe the evolution of the microbiome an experimental approach based on bacterial genus presence or absence and cluster analysis was applied. A list of identified bacteria was created and codified 1/0 patterns were created for each sample (Supplementary Fig. 1). The presence or absence of each bacterial genus was recorded for each sample, to obtain a 1/0 codified pattern. Distance matrix was calculated by using Phi of Pearson Coefficient and used to perform cluster analysis. In particular, complete-linkage dendrograms were generated for each family. Two-dimensional scaling plots (2DS) were depicted for each family, in order to describe the shape and the change of microbiome composition through time. The mean diversity, evaluated as the averaged distances within samples belonging to each family, was assessed at each time point and it was compared to the starting time point (birth). The area covered by the samples belonging to the same family at each time point was calculated (polyarea function embedded in the pracma R package, https://github.com/cran/pracma). The within-family diversity values for each time point were compared as well as the areas of polygons obtained by 2DS analysis (Wilcoxon rank sum exact test) to the first experiment time point (birth). All the statistical analyses were performed using R statistical software.

## Supplementary Information

**Additional file 1. **

## Data Availability

The datasets generated during and analyzed during the current study are not publicly available due to internal regulations but are available from the corresponding author on reasonable request.

## Abbreviations

ACD: Appenzeller Cattle Dog
BA: Blood Agar
BEA: Bile-Esculin Azide Agar
HIB: Heart Infusion Broth
LR: Lagotto Romagnolo
MALDI-TOF MS: matrix-assisted laser desorption/ionization mass spectrometry
SD: Standard Deviation
2DS: two-dimensional scaling

## References

[1] Senn V, Bassler D, Choudhury R, Scholkmann F, Righini-Grunder F, Vuille-Dit-Bile RN (2020). Microbial Colonization From the Fetus to Early Childhood A Comprehensive Review. Front Cell Infect Microbiol.

[2] Nuriel-Ohayon M, Neuman H, Koren O (2016). Microbial Changes during Pregnancy, Birth, and Infancy. Front Microbiol.

[3] Martín R, Langa S, Reviriego C, Jimínez E, Marín ML, Xaus J (2003). Human milk is a source of lactic acid bacteria for the infant gut. J Pediatr.

[4] Murphy K, Curley D, O'Callaghan TF, O'Shea CA, Dempsey EM, O'Toole PW (2017). The Composition of Human Milk and Infant Faecal Microbiota Over the First Three Months of Life: A Pilot Study. Sci Rep.

[5] Solís G, de Los Reyes-Gavilan CG, Fernández N, Margolles A, Gueimonde M (2010). Establishment and development of lactic acid bacteria and bifidobacteria microbiota in breast-milk and the infant gut. Anaerobe.

[6] Lyons KE, Ryan CA, Dempsey EM, Ross RP, Stanton C (2020). Breast Milk, a Source of Beneficial Microbes and Associated Benefits for Infant Health. Nutrients.

[7] Zimmermann P, Curtis N (2020). Breast milk microbiota: A review of the factors that influence composition. J Infect.

[8] Levin AM, Sitarik AR, Havstad SL, Fujimura KE, Wegienka G, Cassidy-Bushrow AE (2016). Joint effects of pregnancy, sociocultural, and environmental factors on early life gut microbiome structure and diversity. Sci Rep.

[9] Mesa MD, Loureiro B, Iglesia I, Fernandez Gonzalez S, LlurbaOlivé E, GarcíaAlgar O (2020). The Evolving Microbiome from Pregnancy to Early Infancy A Comprehensive Review. Nutrients.

[10] Khine WWT, Rahayu ES, See TY, Kuah S, Salminen S, Nakayama J (2020). Indonesian children fecal microbiome from birth until weaning was different from microbiomes of their mothers. Gut Microbes.

[11] Fallani M, Amarri S, Uusijarvi A, Adam R, Khanna S, Aguilera M (2011). Determinants of the human infant intestinal microbiota after the introduction of first complementary foods in infant samples from five European centres. Microbiology (Reading).

[12] ZakošekPipan M, Kajdič L, Kalin A, Plavec T, Zdovc I (2020). Do newborn puppies have their own microbiota at birth? Influence of type of birth on newborn puppy microbiota. Theriogenology.

[13] Rota A, Del Carro A, Bertero A, Del Carro A, StarvaggiCucuzza A, Banchi P (2021). Does Bacteria Colonization of Canine Newborns Start in the Uterus?. Animals (Basel).

[14] Saijonmaa-Koulumies LE, Lloyd DH (2002). Colonization of neonatal puppies by Staphylococcus intermedius. Vet Dermatol.

[15] Gopalakrishna KP, Hand TW (2020). Influence of Maternal Milk on the Neonatal Intestinal Microbiome. Nutrients.

[16] Chastant-Maillard S, Aggouni C, Albaret A, Fournier A, Mila H (2017). Canine and feline colostrum. Reprod Domest Anim.

[17] Martín R, Olivares M, Pérez M, Xaus J, Torre C, Fernández L (2010). Identification and evaluation of the probiotic potential of lactobacilli isolated from canine milk. Vet J.

[18] Guard BC, Mila H, Steiner JM, Mariani C, Suchodolski JS, Chastant-Maillard S (2017). Characterization of the fecal microbiome during neonatal and early pediatric development in puppies. PLoS One.

[19] Vilson Å, Ramadan Z, Li Q, Hedhammar Å, Reynolds A, Spears J (2018). Disentangling factors that shape the gut microbiota in German Shepherd dogs. PLoS One.

[20] Rotimi VO, Duerden BI (1981). The development of the bacterial flora in normal neonates. J Med Microbiol.

[21] Mueller NT, Bakacs E, Combellick J, Grigoryan Z, Dominguez-Bello MG (2015). The infant microbiome development: mom matters. Trends Mol Med.

[22] Allaker RP, Jensen L, Lloyd DH, Lamport AI (1992). Colonization of neonatal puppies by staphylococci. Br Vet J.

[23] Aagaard K, Ma J, Antony KM, Ganu R, Petrosino J, Versalovic J (2014). The placenta harbors a unique microbiome. Sci Transl Med.

[24] Collado MC, Rautava S, Aakko J, Isolauri E, Salminen S (2016). Human gut colonisation may be initiated in utero by distinct microbial communities in the placenta and amniotic fluid. Sci Rep.

[25] Buddington RK (2003). Postnatal changes in bacterial populations in the gastrointestinal tract of dogs. Am J Vet Res.

[26] Reddy KE, Kim HR, Jeong JY, So KM, Lee S, Ji SY (2019). Impact of Breed on the Fecal Microbiome of Dogs under the Same Dietary Condition. J Microbiol Biotechnol.

[27] Kaeberlein T, Lewis K, Epstein SS (2002). Isolating "uncultivable" microorganisms in pure culture in a simulated natural environment. Science..

[28] Perez-Muñoz ME, Arrieta M-C, Ramer-Tait AE, Walter J (2017). A critical assessment of the “sterile womb” and “in utero colonization” hypotheses: implications for research on the pioneer infant microbiome. Microbiome.

[29] Kajdič L, Plavec T, Zdovc I, Kalin A, Zakošek Pipan M (2021). Impact of Type of Parturition on Colostrum Microbiota Composition and Puppy Survival. Animals.

[30] Kuhn G, Pohl S, Hingst V (1991). Investigations on bacteria in the milk of healthy lactiferous bitches. Berl Munch Tierarztl Wochenschr.

[31] Münnich A, Kutzer P, Nattermann H (2000). Aerobic and anaerobic vaginal, milk and oral flora in bitches of a Golden Retriever kennel - the transmission to newborn puppies and relationship to reproductive disorders. Reprod Domest Anim.

[32] FEDIAF. Nutritional Guidelines For Complete and Complementary Pet Food for Cats and Dogs. FEDIAF Org., Bruxelles 2019 [Available from: https://fediaf.org/images/FEDIAF_Nutritional_Guidelines_2019_Update_030519.pdf]. Accessed 10 Nov 2021.

