# Supplementary figures and images for: The evolution of dam-litter microbial flora from birth to 60 days of age
Source: BMC Vet Res. 2022 Mar 11;18:95. doi: 10.1186/s12917-022-03199-3 (PMC8915469; doi:10.1186/s12917-022-03199-3)

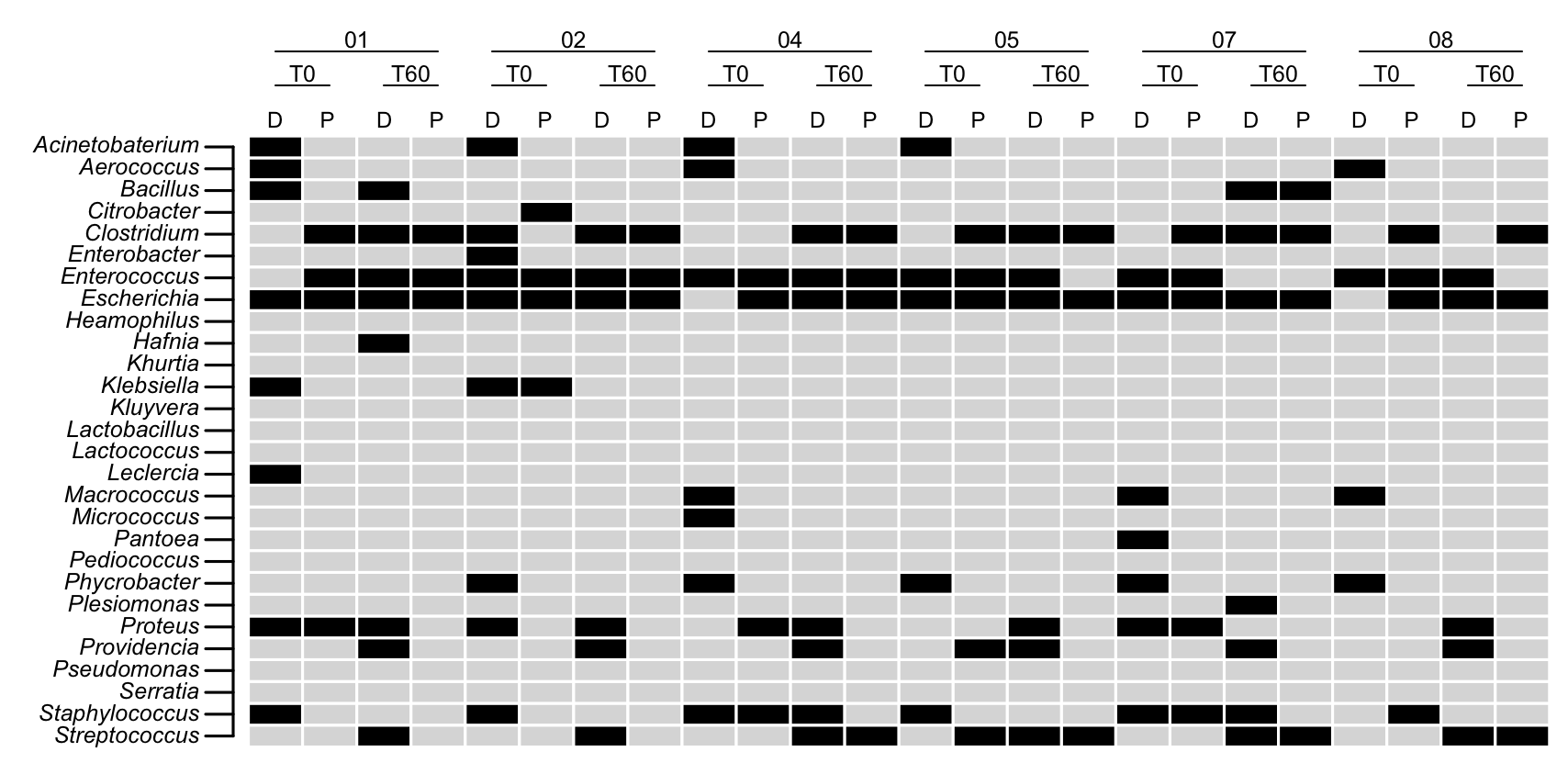

Supplement: Supplementary file 1 — Additional file 1. [file 12917_2022_3199_MOESM1_ESM.tiff]
